# Vine Irrigation through Two Shoot Densities in Flavonoid and Non-Flavonoid Compounds in ‘Tempranillo’ Grapes

**DOI:** 10.3390/plants11101378

**Published:** 2022-05-22

**Authors:** Daniel Moreno, María Victoria Alarcón, David Uriarte, Luis A. Mancha, María Esperanza Valdés

**Affiliations:** 1 Center for Scientific and Technological Research of Extremadura (CICYTEX), Food and Agriculture Technology Institute of Extremadura (INTAEX), Avenue Adolfo Suárez s/n, 06071 Badajoz, Spain; daniel.moreno@juntaex.es; 2 Agricultural Research Center “Finca La Orden-Valdesequera”, Center for Scientific and Technological Research of Extremadura (CICYTEX), Crta. A-V, Km 372, 06187 Badajoz, Spain; maria.alarcon@juntaex.es (M.V.A.); david.uriarte@juntaex.es (D.U.); luisalberto.mancha@juntaex.es (L.A.M.)

**Keywords:** shoot thinning, anthocyanin, flavonol, flavanol, hydroxycinnamic acid, resveratrol

## Abstract

This study aims to analyze the effects of non-limiting irrigation (*I*) vs. rainfed (*R*) through two different shoot densities, high-load (*H*) and low-load (*L*), on vegetative growth, agronomic parameters, flavonoid and non-flavonoid polyphenol substances of cv. Tempranillo grown in a semi-arid climate during three consecutive seasons (2014–2016). Under these conditions, in the 2015 and 2016 seasons, irrigation showed significant increases in berry weight (14.7% and 13.4% in *H* and *L*, respectively, in 2015, and 35.6% and 23.5% in the same treatments in 2016) and yield (66.7% and 48.5 in 2015; 27.9% and 177.5% in 2016). Additionally, a general decreasing trend is observed in anthocyanins with the exception of peonidin derivates, almost all flavonol compounds, cinnamic acid and resveratrol values with different degrees and statistical significance depending on the shoot density of the vines. A slight variation is observed in 2014 in these parameters. On the other hand, no general trends are established either in flavanol compounds or hydroxybenzoic acid. Thus, the effect of irrigation depends on the parameter considered, the shoot density of the vine and the season considered.

## 1. Introduction

A large proportion of vineyards is located in regions with seasonal drought (e.g., Mediterranean-type climates), where soil and atmospheric water deficits together with high temperatures exert large constraints on yield and quality. The increasing demand for vineyard irrigation requires an improvement in water use efficiency.

In arid and semi-arid environments, irrigation is a major tool used to regulate soil water availability to vines. Under these conditions, supplying irrigation to ensure potential vine evapotranspiration increases yield, and sometimes reduces wine quality [1,2,3]. Winter pruning is the most widely used viticultural technique to regulate crop yield and achieve a targeted grape composition, notwithstanding the fact that the number of shoots per vine is not an accurate predictor of harvest yield [4,5]. This technique is often followed by shoot thinning with the removal of fruiting shoots from the vine in order to reduce grape production and plant canopy density [6] and to encourage light and air penetration into the canopy [7,8]; thus, improving the canopy microclimate and grape quality [9,10,11]. Shoot thinning also facilitates more desirable shoot spacing along canes and cordons, and a more even leaf area distribution in the canopy [7,8,12]. Sometimes these conditions can lead to improved bud fertility, fruit set and the mitigation of yield losses. Shoot thinning often increases yield and decreases vegetative growth, leading to a higher ratio of yield to leaf areas, although in other cases, irrigation at 100% of crop evapotranspiration does not adversely affect grape or wine composition [13,14]. In addition, due to the high dependence of fruit quality on various environmental and endogenous factors [15], the overall effect of irrigation might change according to other cultural practices, particularly those affecting the crop level [16,17]. Vines with a higher crop level seem to benefit more from a higher amount of irrigation, both in terms of yield [18] and of fruit composition [19]. This is normally because under high yield, a source limitation for carbohydrates derived from water stress might be more detrimental to proper fruit ripening; hence, negatively affecting fruit and wine quality.

Grapevine quality mainly depends on the primary metabolites (sugars, organic acids and nitrogen compounds) and on the secondary metabolites (phenolics and aromatic substances) [20,21]. Polyphenolic compounds are important secondary metabolites of higher plants that are extensively studied due to their potentially beneficial effects on human health [22]. In the berry, phenolic compounds are localized in skins and seeds, from where they are extracted at different extents during winemaking, significantly influencing different attributes such as the color, flavor, texture, astringency and organoleptic properties of resulting wines [23,24]. These compounds include flavonoids (i.e., anthocyanins, flavonols and flavanols) and non-flavonoids (i.e., hydroxycinnamic acids and stilbenes). The main anthocyanins identified in the species *Vitis vinifera* L. are cyanidin, peonidin, petunidin, delphinidin and malvidin [25]. These anthocyanidins differ from each other in the number and position of the hydroxyl and methoxyl substituents located on the benzene ring. The sugar, which is generally glucose, can be esterified by a phenolic acid, generally caffeic or *p*-coumaric acid (*p*-coumarilated anthocyanins), or acetic acid (acetylated anthocyanins) [26]. Anthocyanidins are synthesized during veraison, which corresponds to the color change [27], and are accumulated in the vacuoles of the first three or four hypodermal cell layers of the berries [28,29] and, in some cases, in the mesocarp and seeds [30,31]. Flavonols are also largely localized in the grape skins, where they are found as flavonol glycosides of quercetin, kaempferol, myricetin and isorhamnetin. Flavan-3-ols, such as catechin and epicatechin, and flavan-3,4-diol dimers, such as B1, B2 and B3, are present in the skin and mainly in grape seeds. Lastly, hydroxycinnamic acids, such as coumaric, caffeic and ferulic acids, and their tartaric esters or diesters caffeoyl tartaric acid, *p*-coumaric acid (coumaroyl tartaric acid) and fertaric acid (feruloyl tartaric acid) are commonly accumulated in berry skin and the flesh of white and red vinifera and non-vinifera varieties [32]. Several studies have shown that the phenolic content in grapes may vary according to varieties, environmental factors and agronomic techniques [33,34,35,36,37,38,39]. With regard to the vine water status, it has been shown that a water deficit improves the accumulation of phenolic compounds, especially anthocyanins [40,41,42] due to direct effects on flavonoid gene expression and metabolism [43].

Color is an important factor for evaluating the quality of red wine, and is linked to the accumulation of anthocyanins in the grape berry skin. Previous works have shown that extraction efficiency from the grape berry into the must/wine also depends on the grape anthocyanin profile [44], as some authors have reported lower extraction yields for coumaroylated anthocyanins [45]. Thus, the full exploitation of the grape potential reached in the vineyard requires the correct management of the winemaking process, particularly the maceration–fermentation stage. The wine industry has turned its attention to assessing anthocyanin extractability [46,47], since grapes rich in anthocyanins at harvest do not usually produce highly colored wines. Therefore, the need for knowing the tendency of the berry skin to yield anthocyanins is evident [48]. However, it is not only the anthocyanin content that is responsible for wine color: it has been reported that co-pigmentation can account for between 30 and 50% of the color in young wines [49]. Co-pigmentation in wine results from molecular interactions between anthocyanin pigments and other organic molecules, called cofactors, forming molecular associations or complexes. The most common cofactors include a variety of compounds, such as phenolic acids, flavonoids and particularly derivatives of the flavonol and flavone subgroups [50]. Knowledge of the polyphenolic profile facilitates the labors of winegrowers to apply the most appropriate techniques for each grape variety, as several works have shown that the different polyphenolic compounds respond differently to viticulture techniques [34,51,52,53,54].

The objective of this study was to investigate, in berries with the same level of total soluble solids, the effect of irrigation in comparison with rainfed vines. Within each irrigation regime, two crop levels were tested, where crop levels were regulated by shoot thinning. The effects of these combined irrigation and crop level treatments on the polyphenol profile of grapes at the same maturation technological stage are analyzed and discussed.

## 2. Results

### 2.1. Meteorological Conditions

The climate of this area is very hot according to the Geoviticulture MCC classification system [55]. Table 1 reflects the meteorological conditions of the three seasons of the trial (2014–2016). The annual mean values of T_Max_, T_Mn_ and T_Min_ were similar during the different years; however, the temperatures and the heat accumulation value, calculated as the growing degree days (GDDs, 2194), reached during the vegetative period (April–August) were slightly higher in 2015 than in 2014 and 2016. Thus, the different rainfalls might have caused variations in bioactive compounds and other parameters. In terms of rainfall, in fact, the years were very different. The annual rainfalls were 491, 310 and 516 mm in 2014, 2015 and 2016, respectively. Therefore, while 2014 and 2016 could be considered normal rainfall seasons, 2015 was a very dry year with values considerably lower than the 489 mm corresponding to the multi-year average (1973–2016). Specifically, the rainfall during the vegetative period reached 88, 60 and 178 mm in those years, respectively. Thus, 2014 was the coldest vegetative season, 2015 the driest and warmest and 2016 the wettest.

### 2.2. Effects of Treatments and Year on Vegetative Growth, Agronomic Parameters and Polyphenolic Content

Table 2 shows the results of the ANOVA applied to the results obtained from the vegetative, agronomical and polyphenolic content of cv. Tempranillo grapes at harvest during the three years of the experiment. The table indicates a high effect of year on all those parameters. Furthermore, worth noting is the significant interaction found in most agronomic parameters, as well as in the content of phenolic substances. Thus, the results were analyzed year by year.

### 2.3. Agronomic Parameters

The vegetative growth (leaf area, LA), cluster number (CN), berry weight (BW), cluster weight (CW), yield (Y) and ratio (LA/Y) at harvest from the 2014 to 2016 vintages are reported in Table 3. Although it was significant only in 2015 and 2016, the same trend was observed in the three seasons of the experiment: irrigation stimulated the vegetative growth and vine leaf area. The percentage increases in *IH* respect to *RH* were 69.05%, 140.91% and 122.86% in 2014, 2015 and 2016, respectively. These increases were higher than those found when comparing *IL* and *RL* in the same years (2.81%, 99.31% and 57.31%, respectively).

On the other hand, increases were found in the values of CN, CW and Y in irrigated treatments in respect to non-irrigated ones in *L* and *H* treatments in all years. Their significance and extent, however, depended on the parameter, crop load, and year considered. In 2014, irrigation did not have a significant effect on the values of any parameters, and in the following years, the values of these parameters were higher (*p* < 0.05) in *IL* than in *RL,* except for the cluster number in 2015. With regard to BW, with the exception of the decrease in *IL* vs. *RL* in 2014, the general trend found was a berry weight increase, which was higher in *I* treatments (*H* and *L*). Due to the importance of berry size for the synthesis and accumulation of phenolic compounds, a decrease observed in the values of all treatments in 2016 compared to the previous years was observed. When analyzed year by year, a decrease in weight of the *IL* berry (2.07 g) compared to the *RL* (2.30 g) stood out. In 2015 and 2016, the *I* berries had higher values (*p* < 0.05) than the *R* berries. The highest increases (35.58%) were found when *IH* and *RH* were compared in the 2016 season. When all treatments were compared, it was observed that the lowest value was reached in *RH* in all years, while the highest was in *RL* in 2014 and in the following years in *IL*. Furthermore, in 2015 and 2016, the values of berries from irrigated treatments were very close, showing the following sequence in those years: *IL* > *IH* > *RL* > *RH*. In respect to yield, the lowest and highest values were found in *IH* and *RL* for all years. Finally, no clear trend was found in the ratio LA/Y.

### 2.4. Polyphenolic Compounds

#### 2.4.1. Polyphenolic Families—Year Effect

**Figure 1:**
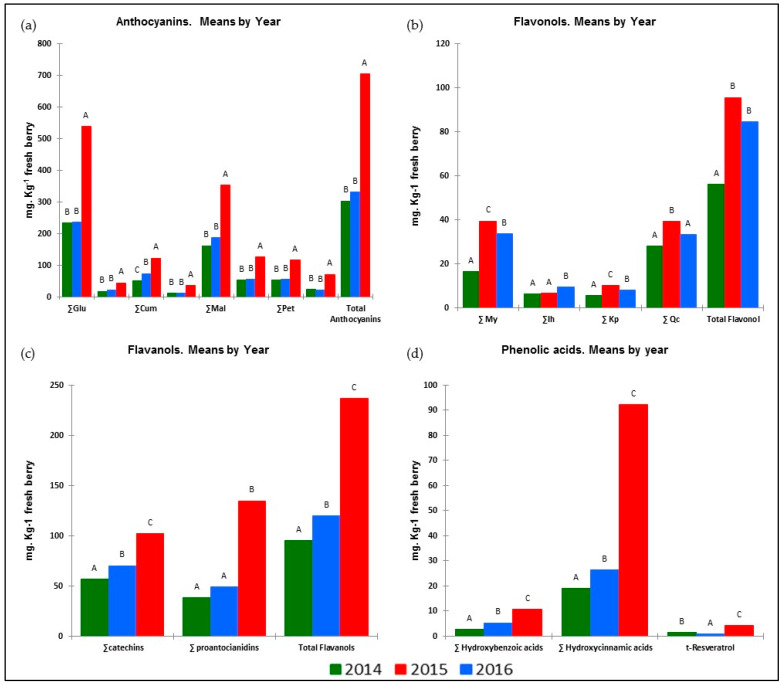
Influence of season on berry phenolic families during the 2014–2016 seasons: (**a**) anthocyanins; (**b**) flavonols; (**c**) flavanols; (**d**) phenolic acids and resveratrol. (For each parameter, means followed by different capital letters are significantly different between treatments, *p* < 0.05, Tukey’s HSD test.)

As Table 3 shows, the different meteorological conditions in the years of the trial had a strong impact on vegetative growth and yield components. All these facts had an impact on the synthesis and accumulation of phenolic compounds. The results presented in Figure 1 show that the response to these factors was similar for all groups belonging to the same phenolic family. It is worth noting that the weather conditions in 2016 contributed to a better biosynthesis and higher accumulation of anthocyanins (ANs), flavanols (FLAVA), hydroxybenzoic (HB) and hydroxycinnamic acids (HA) and resveratrol (tR) in the berries. However, those of 2015 gave the highest values of flavonols (FLAVO) and the lowest values of tR. 

#### 2.4.2. Flavonoid Compounds

##### Anthocyanins

Fifteen anthocyanic compounds were identified, quantified and grouped into anthocyanin monoglucosides (∑Glus), acetyl glucosides (∑Acs) and coumaroyl glucosides (∑Coums) of delphinidin (Dp), cyanidin (Cy), petunidin (Pt), peonidin (Pn) and malvidin (Mv) (Table 4). The total amount of ANs was given in mg of malvidine-3-glucoside. kg^−1^ fresh berries. The results show that, regardless of the season and treatment, Glus were predominant forms, followed by Coums and Acs, and Mv-derived compounds were the predominant anthocyanin-derived substances, while Pt and Cy derivatives were the least abundant. Thus, malvidin glucoside was the major individual anthocyanin compound, and cyanidin acetate and peonidin acetate were the minority compounds in both seasons (data not shown). A similar profile was found in the Tempranillo cultivar in different geographic areas in Spain [33,34,38]. When the effect of irrigation was examined year by year, it could be seen that, in 2014, irrigation decreased the ∑Coums, ∑Acs and ∑Mvs, but it had no impact on the total values in either *H* or in *L* treatments. Furthermore, the decreases were higher in *L* than in *H*. In both *H* and *L* treatments, the irrigation reduced the total values of anthocyanin compounds in the following seasons, except for Pn and Cy, and a significant decreasing trend was found in all anthocyanin derivates. These decreases were greater in 2015 than in 2016, and in *H* in respect to *L* treatments. When all treatments were compared, *IL* had the lowest values of total anthocyanin compounds in all the years of the trial, while the highest corresponded to *RL* in 2014 and to *RH* in the rest of the seasons. 

##### Flavonols Compounds

As shown in Table 5, in all seasons and regardless of the treatment, ∑My and ∑Qc were the predominant flavonol compounds, with myricetin-3-glucoside (MyG) being the most abundant, except in *H* treatment in 2014, in which ∑Kp and ∑Ih were the minor compounds in the profile of cv. Tempranillo.

As with anthocyanins, the effect of irrigation on flavonol compounds in the *H* and *L* treatments was not clear in 2014. For some compounds, a trend opposite to that of the following years was observed and significant increases were found in QcGL and ∑Qc, when *IH* was compared to *RH*. However, in 2015 and 2016, irrigation had a clear impact in all flavonol compounds with significant decreases of more than 30%, in both *H* and *L*. Moreover, the effect of irrigation was similar for both loads and, in general, the percentage decrease for each compound was similar in the *H* and *L* treatments. The decreases in MyG ranged from 36.92% (*p* < 0.01, *RL* vs. *IL* in 2015) to 56.83% (*p* < 0.001, *RH* vs. *IH* in 2016) and in total flavonols from 36.94% (*p* < 0.001, *IL* vs. *RL* in 2015) to 53.23% (*p* < 0.001 in the same comparison in 2016). In the latter two years, *RH* and *RL* values were very close and higher than those found in *IH* and *IL*.

##### Flavanol Compounds

Table 6 shows the flavanol profile of grapevine cv. Tempranillo. Regardless of the season and treatment, (−)-Epigallocatechin (EGC) and Pro B1 were the most abundant catechin (CAT) and proanthocyanidin (PRO), respectively, while Pro A2 was the least abundant flavanol. 

Overall, the response of these compounds to the water status was low and inconsistent in 2014 and 2015. In 2014, a decrease in catechin (CAT) compounds was observed in only *IL* vs. *RL*, and no changes in proantocyanidin (PRO) values were found in the *H* and *L* treatments; in 2015, the only change was an increase in CG in *RL* vs. *IL*. Finally, in 2016, significant decreases and increases in CAT and PRO compounds were found, but without following any specific pattern. Thus, in this season, there were no changes in total CAT, i.e., decreases (in *RH* vs. *IH*) and increases (in *IL* vs. *RL*) in total PRO. Finally, the highest and lowest total FLAVO values were achieved in *RH* and *RL*, respectively. When comparing all treatments, no clear trend could be established. Only a slight tendency to higher values of CAT in *H* compared to *L* treatments was observed in the last two seasons.

#### 2.4.3. Non-Flavonoid Compounds

Table 7 shows the non-flavonoid polyphenolic profile identified and quantified in cv. Tempranillo grapes grouped into phenolic acids (hydroxybenzoic (HB) and hydroxycinnamic (HA)) and stilbenes (tR). Among the phenolic acids, HA was the predominant one and, more specifically, cinnamic acid (CIN) in all treatments, with the exception of coumaric acid (COU) in *I* treatments in the 2015 season. The highest values of these compounds were observed in the 2016 season compared to 2014 and 2015. 

The effect of irrigation on these compounds differed according to the year, crop load and group considered. In respect to GA, no effect was recorded in 2014, an increase in *IH* vs. *RH* was seen in 2015 and, finally, in 2016, there were significant decreases in grapes from irrigated vines compared to rainfed vines to similar degrees in both crop loads. Irrigation decreased the total HA in the *L* load in 2014, although in 2015, it increased in *H* treatments. Finally, in the 2016 season, irrigation decreased the contents of both *H* and *L* grapes. 

Irrigation did not affect t-resveratrol (tR) values in the 2014 season, and in the following season, it decreased with different significance and extent: in 2015, a decrease of 40.37% was recorded in *IH* vs. *RH,* and in 2016, decreases of 3.99 and 3.42% (*p* < 0.01) were reported in *RH* vs. *RH* and in *Il* vs. *RL*. 

#### 2.4.4. Classification of Treatments 

**Figure 2:**
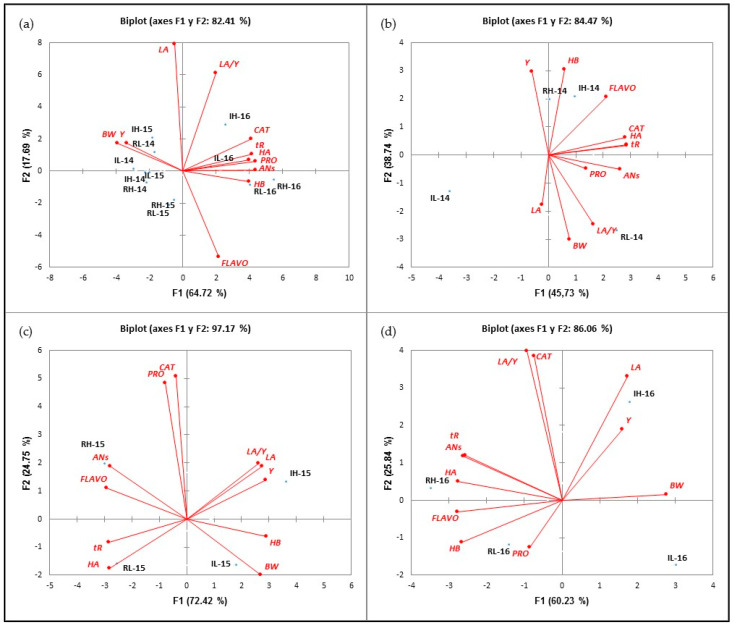
Classification of the treatments analyzed in the function of F1 and F2 for all the analyzed variables during the 2014-2016 (**a**), 2014 (**b**), 2015 (**c**) and 2016 (**d**) seasons.

The principal component analysis (PCA) was used to classify the different treatments in terms of values of vegetative (LA), agronomic (Y and BW) and phenolic (AN, FLAVO, CAT, PRO, HB, HA and tR) parameter values. The first PCA (Figure 2a) was performed with data from the 2014, 2015 and 2016 seasons. The two principal components, F1 and F2, explained 82.41% of the total variance (64.72% and 17.69%, respectively). This axis differentiated the samples from the 2014 and 2015 seasons, located on the negative side of F1 and correlated with BW and Y with the 2016 samples, situated on the positive axis of F1 and associated with higher values of all polyphenol families. The second PCA was performed with the data from the 2014 season. This PCA accounted for 84.47% of the total variance (45.73% and 38.74%, in F1 and F2, respectively). According to Figure 2b, three groups could be distinguished: the first one included *HI*-14 and *RI*-14, as treatments associated with the highest concentrations of FLAVO and HB; next, the *L* treatments could be distinguished: *RL*-14 sited in the positive side of F1 and the negative side of F2, respectively, and correlated with the highest of BW and LA/Y values; and finally, *IL*-14 located in the negative side of F1 and F2. 

The third PCA was performed with the values obtained in 2015. The PCA discriminated the four treatments. Figure 2c shows that the first two principal components (F1 and F2) explained 97.17% of the total variance (72.42 and 24.75%, respectively). *RH*-15 and *RL*-15 were located on the negative side of F1, while *IH*-15 and *IL*-15 were on the positive side of the same axis. The *RH*-15 treatment strongly correlated with ANs and FLAVO, while the *RL*-15 treatment was correlated with tR and HA. *IH*-15 and *IL*-15 treatments were sited in the positive side of F1. Both correlated with higher values of vegetative and agronomic parameters, more specifically, *H*-15 with Y, LA and LA/Y and *IL*-15 with BW. On the other hand, it was observed that both *H* treatments were positioned in the positive side of F2 Thank you, the translation is adequate (defined by CAT and PRO), while the *L* treatments were in the negative side of the same axis. Finally, the fourth PCA was performed with the samples from the 2016 season. As Figure 2d reflects, four groups could be distinguished: two of them (*RH* and *RL*-16) in the negative side of F2 (60.23% explanation of variance) and the rest on the positive side of this axis. On the positive side of F1, *IH*-16 and *IL*-16 were located. As observed in 2015, *R* treatments correlated with higher values of phenolic compounds than *I* treatments, and F2 distributed the treatments according to their crop load, so that *H* treatments were sited on the positive side of F2, while *L* was on the negative side.

## 3. Discussion

### 3.1. Impact on Agronomic Parameters

Climate conditions are particularly important for grapevine growth. Heat, drought and light intensity are just some environmental stress factors that dramatically affect grape development, primary and secondary metabolism and, consequently, the final content of polyphenols in berries [56] Today, irrigation is widely applied in vineyards located in very hot areas, such as the one where this study was carried out. Irrigation should be optimized to achieve the best results of yield and berry quality at harvest. However, the results of irrigation application depend on a multitude of factors: some are permanent, such as terroir and cultivar, others are not manageable, as is the case with seasons, and, finally, others can be modified, such as the amount and timing of irrigation waters, and agronomical practices such as the crop load and crop level [3,10,11,19,57]. Generally, the number and size of grape clusters formed during grape development determine harvest yield, which is influenced by several key stages of vine phenology and seasonal conditions [58]. 

From the results of the present investigation, it is noteworthy that in each of the years of the trial, the reduction in shoot numbers achieved via early shoot thinning did not affect vine capacity, given as the total leaf Area (LA). Thus, the LA in *H* (high crop load) vines was similar to non-thinned vines (low crop load, *L*). These results were in agreement with previous studies carried out in the field on ‘Cardinal’ [59], ‘Chardonnay’ [60] and ‘Sangiovese’ [61], in which full vegetative growth compensation was achieved in all the years in vines with a low crop level (*L*) by an increase in the vigor of individual shoots manifested as a similar LA to high crop level vines (*H*).

It has been reported that water deficits reduce berry size and yield, and some studies have even shown that the decreases are linearly related to decreases in stem water potential [62]. In a recent meta-analysis, Mirás-Avalos and Intrigliolo [63] found that this relationship was variety-dependent. In this regard, Girona et al. [64] reported that ‘Tempranillo’ berry quality demonstrated great phenological sensitivity to water stress. According to previous work carried out on this cultivar [65,66,67], irrigation caused an increase in LA, BW, CW and Y in *H* (high crop load) and in *L* (low crop load). It was observed that the extent of the effect varied with the cultural practices, affecting the crop level, as has been demonstrated in several works [16,17,66]. Vines with a higher crop level seemed to benefit more from a higher amount of irrigation both in terms of yield and of fruit composition [3,66]. In this work, no generalized trend was found for yield; neither the ratio LA/Y nor the largest increases in respect to *RH* and *LH* were found in *IH* or *IL* (in %), respectively, depending on the seasons. However, every year, the increase (in %) in vine LA and BW in *I* respect to *R* vines was more noticeable in *H* than *L* vines (decreases were even found in *IL* in respect to *RL* in 2014). The relation between grape weight and phenolic substance accumulation has been investigated by several authors [68,69,70], and it has been considered that smaller berries accumulate more phenolic compounds [71,72,73]. Thus, considering the influence of berry weight on the synthesis, accumulation and concentration of phenolic substances at harvest, the effect of irrigation on berry weight in this work is of great relevance. 

### 3.2. Incidence for the Year

It is known that the values of polyphenols in berries from a specific cultivar and vineyard and similar TSS are highly dependent on the season [32,38,74,75,76]. The highest values of ANs, FLAVA, HB, HA and tR were reported in 2016 (the rainiest year). Vilanova et al. [39] showed that the composition of grapes was more affected by vintage than by geographic area. These results disagree with those of previous studies carried out in wetter areas, where the highest amounts of polyphenols were detected in the driest years [38,39]. It is possible that the spring rainfall during this last season contributed to a higher synthesis and accumulation of these compounds in the conditions of our trial. On the other hand, these rainfalls caused an oidium (*Uncinula necator*) infestation, which resulted in a large decrease in yield. Moreover, grapes were smaller in 2016 than in the previous seasons. As the summer temperature rose to atypical values, the anthocyanin biosynthetic genes were downregulated, reducing berry skin anthocyanin biosynthesis [77]. For instance, Tarara et al. [78] showed that high temperatures are associated with decreases in grapevine Dp, Cy, Pt and Pn-based anthocyanin contents, but found no influence on Mv derivative concentrations. However, during 2015, the drier and warmer growing season promoted a higher synthesis and accumulation of FLAVO. 

### 3.3. Incidence of Irrigation through Different Shoot Densities in the Phenolic Families

Several specialized (also known as secondary) berry metabolites strongly respond to abiotic stressors, such as water deficits. Among these metabolites, polyphenols are very important, since they contribute to a large extent to grape and wine color, astringency and quality. Of the different classes of polyphenols present in grape berries, the most important are flavonoids (anthocyanins, flavonols and proanthocyanidins (also called condensed tannins)) and stilbenes. Previous works analyzing the effect of water stress on polyphenols report that their effect depends on the season, weather conditions, grapevine variety, magnitude and timing of water deficit and cultivar techniques applied in the vineyard [53,79,80,81,82,83].

As cited above, many works describe the relationship between berry weight and the values at harvest of components which determine must quality. In the present work, the values of polyphenol families at harvest were generally related to the effect on berry weight. This would explain, in part, the low effect of the treatments in the first year of the trial, and the different effects of irrigation on *H* and *L* vines. In this last regard, Walker et al. [84] and Matthews and Nuzzo [85] concluded that the resultant winemaking traits of large or small berries depended more on the factor modifying the weight of the berry (variety, water deficit, etc.) than on the size itself. In this sense, the irrigation on *H* and *L* crop loads caused differences (*p* < 0.01) in stem water potential mean values of vines in 2014–2016 (−0.84 MPa and −0.74 MPa, respectively), yield, LA and ratio LA/Y, and all these factors caused differences in the synthesis and accumulation of phenolic compounds during the vegetative period and, in consequence, in values at harvest. Jackson and Lombard [15] demonstrated that within each genetic material and terroir, vine water status and vine yield (i.e., crop level) or the balance between the sources (vine capacity to produce photoassimilates) and sink demand (grape yield) (i.e., crop load) are probably the major determinants of vineyard performance and fruit composition. Our study focused particularly on profiling the different phenolic families. This is of considerable relevance, as not all phenolic compounds have the same importance in regard to the intensity, tone and color stability of wines [49]. Furthermore, the results achieved show the different sensitivity of the different phenolic compounds to those factors.

In most works, irrigation decreased the total anthocyanin concentration in grapes [53,86,87,88,89,90]. This decrease was caused, on the one hand, by the indirect effect of berry size variation cited above, and, on the other, by the enhanced accumulation of anthocyanins through the stimulation of anthocyanin hydroxylation, probably by upregulating the gene encoding the enzyme F3050H [47,91]. In addition, F3050H transforms Cy and Dp into Pn, Pt and Mv [92,93]. Our work confirmed the previous findings by Castellarin et al. [93] and Deluc et al. [88]. According to these authors, the principal anthocyanins synthesized in the berries under water deficits are Pn-3-O-b-glucoside and Mv 3-O-b-glucoside, because the methoxylation of Dp to produce its derivate Pt rarely occurs. In our work, both in *H* and *L* vines, a large and significant effect was observed in Mv in 2014 (−14.91 and −30.69%, respectively), 2015 (−44.62 and −40.99%, respectively) and in 2016 (−30.09 and −29.18%, respectively). In terms of the Pt, an irrigation-induced decrease was also observed at both crop load levels in 2015 (−46.69 and −31.84% in *H* and *L*, respectively) and 2016 (−19.89 and −18.54% in *H* and *L*, respectively). This latter finding suggests that, under our conditions, the methoxylation of Dp may have occurred in non-irrigated vines. Moreover, according to [78], high temperatures are associated with decreases in grapevine delphinidin, cyanidin, petunidin and peonidin, but they found no influence on malvidin derivatives.

In *V. vinifera* L., ANs are present as mono-glucoside forms of methoxylated and/or hydroxylated anthocyanidins. The number and type of substituents in the B ring of the AN molecule identify different ANs: Cy and Pn with two substituents (3′-substituted), and Dp, Pt and Mv with three substituents (3′,5′-substituted) [76]. Acylated derivatives are considered to be among the most stable compounds [48]. Furthermore, it is known that Mv-3-glucoside is a less reactive AN than Pn-3-glucoside [76], which is known for being highly reactive [94]. On the other hand, the adjacent hydroxyl groups of o-diphenols are more sensitive to enzymatic oxidation (except for laccase) and non-enzymatic oxidation (catalyzed by copper or iron ions) to produce o-diquinones, or even o-diphenol dimmers. Therefore, Cy, Dp and Pt, which contain the o-diphenol structure on the B ring, are more sensitive to oxidation. However, neither Mv nor Pn possess ortho-positioned hydroxyl groups, which results in their comparatively higher resistance to oxidation [95]. Thus, the AN composition of the grape determines the stability of the color of future wine, and the characterization of the AN profile would presumably permit to know which varieties have a more stable color than others, given that their stability is different. 

However, it is not only the anthocyanin concentration and profile that is responsible for wine color; co-pigmentation phenomena can account for between 30 and 50% of color in young wines [49]. Some authors suggest that the co-pigmentation reactions of anthocyanins are the first phase in the formation of stable polymeric pigments during wine aging [96]. Co-pigmentation in wine results from molecular interactions between AN pigments and other organic molecules, called cofactors, forming molecular associations or complexes. The most common cofactors include a variety of compounds, such as phenolic acids, flavonoids and, particularly, derivatives of flavonol and flavone subgroups [50]. In the co-pigmentation phenomenon, which contributes to the stabilization of the color of red wines [49,75], Baranac et al. [97] reported that flavonol substances were among the best co-pigments, especially quercetin [98]. The profile found was similar to the ‘Tempranillo’ grapes reported in previous works with Qc and My-based flavonols, MyG, QcG and QcGR, dominating [33,34,99]. Given the importance of these phenolic substances on the stability and intensity of the color of red wines through co-pigmentation phenomena, the study and monitoring of agronomic factors and viticultural practices that increase their content and improve the cofactor concentration are major objectives for the production of high-quality wines, especially in terms of their color. Studies have shown that a water deficit has a moderate effect on flavonol synthesis, and the effect of the irrigation application period is almost negligible [92,100]. When Cabral et al. [101] investigated the impact of deficit irrigation on grapevine cv. Touriga Nacional during three seasons in the Douro region, they observed increases in KpG, My-hexoside, QcG, KpR and QcGR in two of the three vintages, while MyG decreased only in two of them. Gamero et al. [83], in wines elaborated from vines grown in similar edaphology and climatic conditions to those in the present work, reported a different irrigation effect depending on whether the wines were created from thinned or control vines. However, little or no change was noticed for other flavonoid compounds. The synthesis of these substances is light dependent, so the higher leaf area observed in *IH* and *IL* treatments may also have contributed to the decrease. Additionally, in this work, a similar response of ANs to irrigation was found in FLAVO compounds. This may be because flavonol biosynthesis is closely related to that of anthocyanins [102] (Jeong et al., 2006), even though it has been suggested that those phenolic family compounds share the same biosynthetic enzymes [91,100]. On the other hand, flavonol occurrence can be considered as a biomarker for a sun exposure regime achieved in a bunch area within the canopies following microclimate manipulation management. LA and the LA/Y ratio were higher in *H* than in *L* in 2015. Thus, as expected, the decrease in FLAVO was greater in *H* than in *L*. 

The major flavonoids synthesized in the grapevine berry, anthocyanins and tannins (also known as proanthocyanidins), strongly impact the quality of red wines via their contributions to wine color and astringency [103,104,105]. In this work, in contrast with anthocyanin and flavonols, the differences between treatments were rarely significant, and a clear trend caused by irrigation was not observed in catechins or proantocianidins, regardless of the crop level. The impact of irrigation on these substances still remains unclear, as contrasting results have been reported among studies [43,90,106,107]. The pre- and post-veraison application of water deficit increased proanthocyanindin levels in Syrah and Cabernet Sauvignon berries, but only transiently, and at harvest, no differences were observed [43,106]. In grapes from cv. Graciano grown in plastic pots, irrigated with sustained deficit irrigation (SDI), Niculcea et al. found registered decreases in FLAVO and increases in CA in respect to non-irrigated grapes [40]. In Tempranillo vines grown in the same edaphoclimatic conditions as the present work, Gamero et al. [83] found increases in Pro B1 and Pro B3 dimers in irrigated grapes. When regulated deficit irrigation (RDI) was applied to ‘Monastrell’ grapevines grown in eastern Spain under semiarid conditions, the content of EC in these grapes was mainly flavanol, and epicatechin-3-gallate decreased [108]. Thus, according to Bucchetti et al. [79] in their work with Merlot, our results with Tempranillo indicate that tannin accumulation is less sensitive to water deficits than anthocyanin accumulation, and is largely unaffected by late-season water deficits. The different methods used to determine these phenolic substances could be in part the cause of these results. Moreover, a gene expression study undertaken by Zarrouk et al. demonstrated differential expression during the grape berry development of the ANR gene in grape seeds and a slight downregulation under water stress (cited by [109] of these substances.

Regarding phenolic acids, previous works report different profiles and effects of irrigation depending on the cultivar considered. In their work, cited above, Niculcea et al. [41] found that t-caftaric acid was the main HA, accounting for 55% in Tempranillo and Graciano in well-watered plants, in respect to the irrigation effect, while in Tempranillo, the SDI treatment resulted in increased t-caftaric and caffeic acids, which in Graciano did not alter the total HA at harvest, but its individual composition was modified. Thus, the SDI treatment reduced the concentrations of t-caftaric, c-coutaric and t-coutaric and increased the concentrations of coumaric and ferulic acids. In their triannual work, Cabral et al. registered increases and decreases in gallic and coutaric acids in berries irrigated with 30% and 70% ETc depending on the season considered [101]. Finally, gallic acid was the most abundant phenolic acid in ‘Monastrell’ grapes, and only the content of total hydroxybenzoic acids and trans-fertaric acid was affected by the watering regime, increasing in grapes from rainfed grapevines in respect to those in RDI [108]. Valdés et al. [110] found decreases in hydroxycinnamic acids in berries in respect to early defoliated vines. These authors explained these results on the basis of the lower canopy porosity and decreased cluster exposure of the defoliated vines. Thus, the decreases in these substances in *IH* and *IL* grapes vs. *RH* and *RL* could be explained by the increase in LA in irrigated vines. However, the value of the LA increase did not correlate with the decreased values of these acids. These acids react with monomeric anthocyanin-forming pyranoanthocyanins, which are more stable compounds [111] and, thus, stabilize wine color. Therefore, in addition to winemaking techniques, viticultural practices that increase the content of these substances in the berry must be applied. In this sense, irrigation would not be a recommendable technique.

Generally, stilbenes are considered as phytoalexins, and their formation in grape leaves correlates with disease resistance. It accumulates mainly in the grape skin and seeds of red and white grapes at a wide range of concentrations, depending on biotic and abiotic conditions [112]. Resveratrol is the most bioactive stilbene in grapevines [113] and its synthesis is catalyzed by stilbene synthase (STS) [114]. The highest values of tR were found in 2016, and in this season, the vineyard was infected by oidium. In our work, a general, significant decrease in tR with similar extent was found in *H* and *L* treatments in 2015 and 2016. However, conflicting results have been reported in the literature regarding the effect of irrigation on this substance. While a short drought effect was observed in the tR concentration in grape berry skins in the Barbera cultivar [115] and no significant differences were found by [108] in ‘Monastrell’ grapes, a substantial increase in STS was observed in the Cabernet Sauvignon cultivar [100]. According to the work of Molero, PCAs demonstrated that seasons had a great effect on polyphenol values under our conditions [83,116]. The season effect was even greater than treatment effect. When the PCA plots were examined year by year, anthocyanin and flavonol values were associated with low values of berry weight and yield. Thus, the highest berry weight and lowest antocyanin values were observed in I treatments. Molero et al. [117] reported different values of anthocyans in rainfed and irrigated treatments, but they did not differentiate between *H* and *L* treatments. In this work, Cat and Pro permitted a good separation between *H* and *L* treatments.

## 4. Materials and Methods

### 4.1. Plant Material and Experimental Layout

The present study was conducted from 2014 to 2016 at an experimental vineyard of cv. Tempranillo (*V. vinifera* L.) in Badajoz, Extremadura (western Spain) (lat. 38°51′ N; long. 6°40′ W; elevation 198 m asl) planted at 3333 plants ha^−1^ and trained on a double Royat cordon system as vertical shoot positioning oriented east–west. Vines were spaced 1.20 m within the row and 2.50 m between rows. Soil was loam to sandy loam texture and >2 m deep. 

The vineyard was drip-irrigated with pressure-compensating emitters of 4 L.·h^−1^ located in a single row 60 cm apart. Irrigation was managed uniformly, as follows: water consumption was calculated with a weighing lysimeter located at the experimental vineyard [116]. Irrigation started when the stem water potential (SWP) reached a level of −0.6 MPa [118,119]. The SWP measurements were determined at midday using a pressure chamber (Model Soil Moisture Corp, Santa Barbara, CA, USA).

### 4.2. Treatment Application

The experimental design was a split plot with four replicates. Irrigation was the whole plot factor, and crop load the subplot factor. Two irrigation treatments were established in relation to crop evapotranspiration (ET_c_): rainfed (0% ET_c_) (*R*) and non-limiting irrigation (100% ET_c_) (*I*) throughout the season. The value of ET_c_ was determined with a weighing lysimeter installed in the experimental vineyard. Initially, the crop load was adjusted by winter pruning, and, then, within each irrigation regime, two different shoot densities were adjusted by pruning: high crop load (*H*) (12 shoots per vine) and low crop load (*L*) (6 shoots per vine) at stage 12 (phenological development stages according to [120]). Both practices were combined and, thus, four experimental treatments were established: *RH*, *RL*, *IH* and *IL*. Therefore, the study was carried out in 16 experimental plots, each one consisting of 6 rows of 18 vines (108 vines per experimental plot).

### 4.3. Environmental Conditions

Meteorological data were obtained during the experiment from an agro-climate station (Network of Extremadura Advice to Irrigation; REDAREX) located 100 m from the vineyard. We obtained agrometeorological data from a station close to the vineyard (100 m) with the characteristics described in [121]. Growing degree days (GDDs) were calculated using 10 °C as the base temperature, recorded during the vegetative–productive period [122,123]. Maximum (T_Max_), minimum (T_Min_) and mean temperatures (T_Mn_) and rainfall were registered (Table 1). Mean annual values over 43 years (1973–2016) from REDAREX data base: T_Max_: 24.05 (°C); T_Mn_: 18.04 (°C); T_Min_: 10.60 (°C); Rainfall 488.8 mm.

### 4.4. Agronomic Determinations

When vegetative growth ceased, the mean leaf area per shoot was estimated destructively by measuring the area of all leaves of 10 shoots in each treatment using a LAI-Licor 3100 canopy analyzer (LI-COR Inc. Lincoln, NE, USA). This average leaf area per shoot was multiplied by the total number of shoots of 10 marked vines per experimental plot (40 vines per treatment) to estimate the total leaf area per vine (LA).

Yield at harvest was calculated by weighing ten control vines for each experimental plot, in accordance with [67]. All clusters per vine were weighed and counted in 10 marked vines of each experimental plots to determine the number of clusters and the yield per vine. Leaf area and yield relationship was estimated as the ratio between LA and yield in each of the 10 marked vines in each experimental plot.

### 4.5. Grape Samples

Grapes (*V. vinifera*, L. cv. Tempranillo) were collected at harvest from CICYTEX (Badajoz, Spain) vineyard during 2014, 2015 and 2016 growing seasons. To improve the physiological homogeneity of the different samples, berries were picked from the top, central and bottom parts of the cluster, following a zigzag path between two marked rows of 18 vines. These berries were calibrated according to their density [124]. Density was estimated by flotation of berries in seven different salt solutions from 115 to 175 g·L^−1^ NaCl. Total soluble solids were measured on the several density ranges with a digital ATR ST plus refractometer (Schmidt + Haznsch, Berlin, Germany). At harvest, the berries selected were those which floated between 150 and 170 g·L^−1^, (TSS: 22.5–24.5 °Brix). Berry weight was determined according to the official methods of the International Organisation of Vine and Wine [125]. 

### 4.6. Extraction of Phenolic Compounds and Determination of Total Phenolic Content

Phenolic compounds were extracted from grape berries following the methodology previously described by [126] with some modifications. A total of 300 g of selected berries was crushed and homogenized in a blender for 1 min (speed 3, Worwek Model TM-31, Hamburg, Germany). Of the homogenate obtained, 50 g was macerated with 50 mL oxalic acid buffer 0.3 M (pH 1.00) during 16 h at 22–24 °C, and then centrifuged at 21,952× *g*, 4 °C for 10 min (Allegra 25R Beckman Coulter, Brea, CA, USA). 

### 4.7. Analysis of Phenolic Compounds by HPLC 

HPLC separation, identification and quantification of phenols were performed on an Agilent 1200 Series system (1200 LC; Agilent Technologies, Palo Alto, CA, USA) equipped with a degasser, quaternary pump, column oven, 1290 infinity autosampler, UV–VIS diode-array detector (DAD) and the Chemstation software package for LC 3D systems (Agilent Technologies) to control the instrument and for data acquisition and data analysis. Separation was performed in a Kromasil^®^ column 100–5-C18 250 × 4.6 mm, (Akzonobel, Bohus, Sweden). The supernatant of samples previously obtained was filtered (0.20 µm, Chromafil PET 20/25, Macherey-Nagel, Düren, Germany) and injected directly into the HPLC. 

For identification and quantification of compounds, the analysis was carried out as described in [127]. The column was maintained at 40 °C. The mobile phase consisted of a gradient mixture of a solvent A (0.85% phosphoric acid solution) and solvent B (acetonitrile), with a flow rate of 1 mL.min^−1^. 

The anthocyanins (ANs) present in extracts were identified in the monoglucoside forms (∑Glu) of delphinidin (Dp), cyanidin (Cy), petunidin (Pt), peonidin (Pn) and malvidin (Mv); in the acetylglucoside forms (∑Ac) (DpA, CyA, PtA, PnA and MvA) and in the *p*-coumaroylglucoside forms (∑Coum) (DpC, CyC, PtC, PnC and MvC). The total amount of ANs was given in mg of malvidine-3-glucoside.kg^−1^ fresh berries. The amount of flavanols (∑FLAVA) identified ((+)-catechin (CA), (−)-epicatechin (EC), (−)-catechin gallate (CG), (−)-epigallocatechin (EGC) and the procyanidins A2, B1, B2 and B3) were quantified as mg of (+)-catechin.kg^−1^ fresh berries. Additionally, myricetin (My), quercetin (Qc), kaempferol (Kp), isorhamnetin (Ih) and their 3-glucosides (MyG, QcG, KpG and IhG) and the 3-rutinoside of quercetin (QcR) and kaempferol (KpR), quercetin-3-glucuronide (QcGR) and quercetin-3-galactoside (QcGL) were grouped as flavonols (∑FLAVO) and quantified as mg of quercetine-3-glucoside.kg^−1^ fresh berries. Related to the non-flavonoids compounds analyzed, gallic acid (GA) was grouped as the hydroxybenzoic acid (∑HB), and caffeic (CF), chlorogenic acid (CHL), *p*-coumaric (COU), t-cinnamic acid (CIN) and ferulic acid (FE) were grouped as hydroxycinnamic acids (∑HA) and quantified as mg of caffeic acid.kg^−1^ fresh berries. Resveratrol (tR) was grouped as stilbenes (∑STILs) and quantified as mg of t-resveratrol.kg^−1^ fresh berries.

The diode array detector was employed on four wavelengths: 220 nm for identification of the HB and FLAVA: Gallic acid, (−)-epicatechin, (−)-epigallocatechin gallate, (−)-catechin gallate and procyanidin B1 and B3; 320 nm for HA and STIL; 360 nm for FLAVO and 520 nm for AN. Excitation at 280 and emission at 320 nm were measured by FLD for identification of the following FLAVA compounds: (+)-catechin and procyanidin A2 and B2. Phenolic compounds were identified according to their elution order and retention times of the commercial standards and/or published in previous studies [127,128]. For quantification and calibration of each compound, calibration curves of their respective standards (R^2^ > 0.999) were used (catechin, epicatechin, catechin gallate, epicatechin gallate, myricetin, quercetin and trans-resveratrol (Sigma, St. Louis, USA), malvidin-3-glucoside, procyanidins A2, B1, B2 and B3, quercetin, kaempherol and isorhamnetin glucosides, myricetin-3-glucoside and kaempherol-3-rutinoside (Extrasynthese, Genay, France), quercetin-3-rutinoside, kaempherol, isorhamnetin and quercetin-3-galactoside (Fluka, Buchs, Germany)). Quantification of non-commercial compounds was conducted using the straight calibration compound belonging to the same family, which was next in the order of elution.

### 4.8. Statistical Data Analysis

The effect of treatment, year and treatment × year interaction was evaluated by a one- (treatment) or two-way (treatment, year) ANOVA. For the same crop load level, statistical comparisons between mean values were established with Student’s *t*-test. The mean values of each experimental treatment were compared using Tukey’s HSD test (*p* < 0.05). Finally, phenolic profile data were submitted to principal component analysis (PCA) with the aim of discriminating treatments based on association of the studied variables. The data analyses were performed using XLSTAT-Pro (Addinsoft, Paris, France, 2009).

## 5. Conclusions

Shoot thinning did not alter the effect of irrigation on the vegetative or agronomic parameters: the applied water stimulated vegetative growth, yield and berry weight in the three seasons of the study, both in shoot thinned and un-thinned vines. In regards to the impact on the phenolic content of the berries, it should be noted that for the majority of substances and phenolic groups, a similar behavior in both crop levels was found: irrigation caused in berries from both thinned and unthinned vines decreased in flavonoid compounds such as anthocyanins, flavonols and epigallocatechin, and between non-flavonoid compounds in t-resveratrol and cinnamic acid. However, the degree and statistical significance of the effect depended in some cases on the shoot density of the vine (i.e., anthocyanins and t-resveratrol), and in others it was similar (i.e., flavonols). In addition, it is noteworthy that the season effect was similar in both shoot densities and the impact of irrigation was greater in 2015 and 2016 than in 2014. Our findings are essential to vine growers and should help to decide the most appropriate crop level if full irrigation is applied. In addition, these results provide deeper information on the phenolic profile of cv. Tempranillo. Given the importance of these substances in organoleptic attributes of red wines, detailed knowledge of this profile would enable vine growers to adapt most adequate wine-making techniques to elaborate red wines of high quality.

## Figures and Tables

**Table 1:** Yearly meteorological data of the experimental site in western Spain (Badajoz) from 2014 to 2016. T_Max_: maximum temperature; T_Mn_: mean temperature; T_min_: minimum temperature; GDD: growing degree day (base 10 °C).

	2014				2015				2016			
	T_Max_ (°C)	T_Mn_ (°C)	T_Min_ (°C)	Rainfall (mm)	T_Max_ (°C)	T_Mn_ (°C)	T_Min_ (°C)	Rainfall (mm)	T_Max_ (°C)	T_Mn_ (°C)	T_Min_ (°C)	Rainfall (mm)
January	14.7	10.3	6.3	59.4	13.5	5.9	0.2	34.7	14.7	10.4	6.3	69.3
February	14.8	10.1	5.7	73.0	14.6	8.5	2.9	13.3	15.0	9.8	5.3	46.1
March	18.9	11.9	5.3	28.2	20.1	12.2	4.8	24.8	17.0	10.2	4.0	29.9
April	22.7	16.0	9.9	56.5	22.8	16.0	9.5	33.9	20.2	14.0	10.4	64.8
May	27.2	19.1	11.0	8.1	29.5	21.0	11.5	0.2	23.4	16.8	10.5	94.5
June	29.2	21.5	13.4	16.9	31.9	24.1	15.4	16.2	31.2	22.9	14.1	3.2
July	32.2	23.8	14.8	6.6	34.8	25.7	16.6	9.1	35.6	26.6	17.1	14.9
August	32.6	23.8	14.9	0.0	32.5	24.5	16.3	1.0	35.8	26.6	16.7	0.4
September	28.1	21.3	15.6	47.9	28.4	20.5	13.0	18.2	31.5	22.3	13.4	6.3
October	25.7	18.1	12.3	84.2	23.1	17.1	12.9	109.3	24.8	17.5	11.4	73.7
November	18.1	13.2	8.9	91.1	20.1	11.8	5.9	19.1	17.3	11.2	5.8	74.9
December	13.6	6.8	2.2	18.7	16.7	9.7	4.0	30.5	15.0	8.3	3.3	37.9
Annual	23.1	16.3	10.0	490.6	24.0	16.4	9.4	310.2	23.5	16.4	9.9	515.7
Vegetative–Productive	28.7	20.9	13.3	136.0	30.0	22.0	13.7	78.6	29.6	21.6	13.7	184.0
GDD (°C)	2002				2194				2117			

**Table 2:** Effect of irrigation (treatment) and experimental season (year) and their interactions on values of vegetative, agronomic and polyphenolic content of cv. Tempranillo grapes at harvest.

Significance of Effects	Treatment	Year	Treatment × Year
Leaf area (LA)	***	n.s.	n.s.
Cluster number (CN)	***	n.s.	n.s.
Cluster weight (CW)	***	***	n.s.
Berry weight (BW)	***	***	***
Yield (Y)	***	***	n.s.
Leaf area/yield (LA/Y)	n.s.	n.s.	*
Glucosides (Glu)	***	***	n.s
Acetates (Ac)	***	***	**
Coumarates (Coum)	***	***	**
Cyanidine (Cy)	***	**	**
Delphinidine (Dp)	***	**	n.s
Malvidine (Mv)	***	***	*
Peonidine (Pn)	***	n.s	n.s
Petunidine (Pt)	***	***	n.s
**Total anthocyanins (** **∑An)**	***	***	n.s
(−)-Epicatechin (EC)	***	**	n.s
(+) Catechin (CA)	***	**	n.s
(−)-Catechin gallate (CG)	***	*	n.s
(−)-Epigallocatechin (EGC)	***	***	**
Pro A2	***	n.s	n.s
Pro B1	***	***	***
Pro B2	***	n.s	n.s
Pro B3	***	n.s	n.s
**Total flavanols (** **∑FLAVA)**	***	*	**
Kaempferol-3-Glucoside (KpG)	***	***	**
Kaempferol-3-Rutinoside (KpR)	***	***	**
Isorhamnetin-3-Glucoside (IhG)	**	***	**
Isorhamnetin-3-Rutinosido (IhR)	***	***	***
Quercetin-3-Glucoside (QcG)	***	***	**
Quercetin-3-Rutinosidoe (QcR)	*	n.s	**
Quercetin-3-Glucuronide (QcGR)	***	***	***
Quercetin-3-Galactoside (QcGL)	*	ns	**
Myricetin-3-Glucoside (MyG)	***	***	**
**Total flavonols (** **∑FLAVO)**	***	***	***
Gallic acid (GA)	***	***	***
Caffeic acid (CF)	***	n.s	***
Chlorogenic acid (CHL)	***	**	n.s
p-coumaric acid (COU)	***	***	***
t-Cinnamic acid (CIN)	***	***	***
t-Ferulic acid (FE)	*	n.s	n.s
**Total hydroxycinnamic acids (** **∑HA)**	***	***	***
t-Resveratrol (tR)	***	***	***

Treatment: Irrigation procedures (*RH*, *IH*, *RL*, *IL*) are explained in Section 4.2. ANOVA test significance level. n.s.—not significant; * = *p* < 0.05; ** = *p* < 0.01; *** = *p* < 0.001.

**Table 3:** Influence of irrigation on yield components and vegetative growth at harvest under two crop load levels during the 2014–2016 seasons. *R*: rainfed; *I*: unrestricted irrigation; *H*: high crop load; *L*: low crop load.

Treatment							
Year	Parameter	*RH*	*IH*	% Variation	*RL*	*IL*	% Variation
2014	Leaf area (LA, m^2^·vine^−1^)	1.68 a	2.84 a	69.05	2.85 a	2.93 a	2.81
	Cluster number (CN)	15.18 b	17.05 b	12.32	9.30 a	10.95 a	17.74
	Cluster weight (CW, g)	186.71 a	196.68 a	5.34	194.09 a	234.36 a	20.75
	Berry weight (BW, g)	1.87 a	1.91 a	2.14	2.30 c	2.07 **b	−10.00
	Yield (Y, kg·ha^−1^)	9830 a	10864.2 a	10.52	6093 a	8413.80 a	38.09
	Leaf area/yield (LA/Y, m^2^·kg^−1^)	5.60 ab	4.74 a	−15.36	10.64 a	5.55 b	−47.84
2015	Leaf area (LA, m^2^·vine^−1^)	1.98 b	4.77 a	140.91	1.44 b	2.87 ab	99.31
	Cluster number (CN)	12.83 b	15.33 c	19.49	8.30 a	10.45 a	25.90
	Cluster weight (CW, g)	192.77 c	267.47 **ab	38.75	240.82 bc	313.77 *a	30.29
	Berry weight (BW, g)	1.50 d	1.72 ***b	14.67	1.57 c	1.78 ***a	13.38
	Yield (Y, kg·ha^−1^)	8243.30 b	13744.20 *a	66.73	7295.8 b	10837.50 *ab	48.54
	Leaf area/yield (LA/Y, m^2^·kg^−1^)	5.08 ab	9.53 a	87.60	4.84 b	5.97 ab	23.35
2016	Leaf area (LA, m^2^·vine^−1^)	2.10 b	4.68 a	122.86	1.78 b	2.80 ab	57.30
	Cluster number (CN)	12.73 ab	15.05 a	18.22	8.25 c	11.65 *b	41.21
	Cluster weight (CW, g)	126.61 ab	153.31 a	21.09	90.40 b	175.65 **a	94.30
	Berry weight (BW, g)	1.04 a	1.41 **c	35.58	1.19 b	1.47 **c	23.53
	Yield (Y, kg·ha^−1^)	4937.50 ab	6317.5 a	27.95	2186.70 b	6068.30 *ab	177.51
	Leaf area/yield (LA/Y, m^2^·kg^−1^)	9.26a	12.09 a	30.56	8.83 a	5.15 a	−41.68

*, ** and *** indicate, for the same crop load (*H*/*L*), the significance of irrigation treatment rainfed vines in respect to irrigation at *p* < 0.05, *p* < 0.01 and *p* < 0.001, respectively. For each parameter, means followed by different letters were significantly different between treatments, *p* < 0.05, Tukey’s HSD test.

**Table 4:** Influence of irrigation on concentration of anthocyanins (ANs) compounds (mg. kg^−1^ berry fresh weight) under two crop load levels. *R*: rainfed; *I*: non-limiting irrigation; *H*: high crop load; *L*: low crop load.

		Treatment					
Year	Compound	*RH*	*IH*	% Variation	*RL*	*IL*	% Variation
2014	∑Glu	237.49 a	228.91 a	−3.61	269.46 a	200.90 a	−25.44
	∑Coum	62.00 a	48.54 ** ab	***−15.09***	***60.47 a***	35.92 * b	***−34.62***
	∑Ac	19.35 ab	16.43 * ab	***−21.71***	***21.00 a***	13.73 * b	***−40.60***
	∑Mv	180.67 a	153.73 * ab	***−2.73***	186.05 a	128.95 * b	***−30.17***
	∑Dp	50.59 a	49.21 a	−10.19	63.71 a	44.49 a	−32.86
	∑Pt	55.03 a	49.42 a	22.75	62.76 a	42.14 a	−6.62
	∑Pn	21.30 a	27.97 a	31.31	25.41 a	23.02 a	−9.41
	∑Cy	10.55 a	12.95 a	−14.91	12.09 a	11.29 a	−30.69
	**Total anthocyanin**	318.84 a	293.88 a	−7.83	350.94 a	250.56 a	−28.60
2015	∑Glu	315.17 a	183.97 ** b	***−41.63***	262.11 ab	179.31 * b	***−31.59***
	∑Coum	100.63 a	57.00 ** b	***−49.72***	84.19 a	53.13 ** b	***−42.16***
	∑Ac	31.72 a	15.95 ** b	***−43.36***	26.47 a	15.31 ** b	***−36.89***
	∑Mv	265.06 a	146.79 ** bc	***−43.56***	214.06 ab	126.31 ** c	***−27.21***
	∑Dp	71.10 a	40.13 ** c	***−46.59***	61.74 ab	44.94 * bc	***−34.97***
	∑Pt	74.84 a	39.97 ** b	***−7.06***	63.82 a	41.50 ** b	***6.84***
	∑Pn	24.00 a	18.73 a	−21.96	20.36 a	21.81 a	7.12
	∑Cy	11.48 a	10.67 a	−44.62	11.99 a	12.81 a	−40.99
	**Total anthocyanin**	447.52 a	256.92 ** b	***−42.59***	372.77 ab	247.76 ** b	***−33.54***
2016	∑Glu	622.48 a	532.66 ab	−14.43	525.27 ab	471.09 b	−10.31
	∑Coum	168.12 a	106.65 *** c	***−36.56***	133.12 b	82.34 ** d	***−38.15***
	∑Ac	58.94 a	37.56 *** c	***−36.27***	48.91 b	31.57 *** c	***−35.45***
	∑Mv	461.10 a	322.37 ** bc	***−9.30***	369.98 b	262.01 ** c	***−6.40***
	∑Dp	142.42 a	129.17 ab	−19.89	121.17 ab	113.42 b	−18.54
	∑Pt	139.75 a	111.95 * b	***25.38***	116.39 b	94.81 ** b	***30.41***
	∑Pn	73.01 a	72.34 a	−0.92	67.09 a	72.73 a	8.41
	∑Cy	32.15 b	40.31 * a	***−30.09***	31.67 b	41.30 ** a	***−29.18***
	**Total anthocyanin**	849.53 a	676.87 ** b	***−20.32***	707.30 b	585.00 ** b	***−17.29***

*, ** and *** indicate, for the same crop load (*H*/*L*), the significance of rainfed in respect to irrigation at *p* < 0.05, *p* < 0.01 and *p* < 0.001, respectively. For each parameter, means followed by different letters were significantly different between treatments, *p* < 0.05, Tukey’s HSD test.

**Table 5:** Influence of irrigation on concentration of flavonol compounds (mg. kg^−1^ berry fresh weight) under two crop load levels. *R*: rainfed; *I*: non-limiting irrigation; *H*: high crop load; *L*: low crop load. MyG: myricetin-3-glucoside; QcG: quercetin-3-glucoside; QcR: quercetin-3-rutinoside; QcGR: quercetin-3-glucuronide; QcGL: quercetin-3-galactoside; Qc: quercetin; IhG: isorhamnetin-3-glucoside: IhR: isorhamnetin-3-rutinoside; Ih: isorhamnetin; KpG: kaempferol-3-glucoside; KpR: kaempferol-3-rutinoside; Kp: kaempferol.

Treatment							
Year	Compound	*RH*	*IH*	% Variation	*RL*	*IL*	% Variation
2014	**Trisubstituted**						
	MyG	17.48 a	13.62 a	−22.12	18.91 a	13.34 a	−29.44
	**Disubstituted**						
	QcG	19.41 a	27.36 * a	***40.95***	14.79 a	10.50 a	−28.99
	QcR	0.91 a	0.91 a	−0.24	1.04 a	2.81 a	170.70
	QcGR	3.96 a	3.07 ** a	***−22.53***	4.36 a	1.28 ** b	***−70.69***
	QcGL	2.81 a	3.95 ** a	***40.51***	2.85 a	11.26 a	295.37
	**∑Qc**	27.02 a	35.23 * a	***29.22***	23.04 a	25.79 a	11.16
	IhG	4.99 a	4.03 * a	***−19.22***	5.26 a	2.06 ** b	***−60.93***
	IhR	1.85 a	2.22 a	20.01	1.82 a	2.51 a	37.69
	**∑Ih**	6.84 a	6.25 ab	−8.62	7.09 a	4.57 * b	***−35.54***
	**Total disubstituted**	34.14 a	41.53 * a	***21.64***	30.34 a	30.42 a	0.26
	**Monosubstituted**						
	KpG	0.64 a	0.51 a	−20.26	0.88 a	1.18 a	33.76
	KpR	5.40 ab	6.56 a	21.39	4.86 ab	2.09 b	−57.03
	**∑Kp**	6.04 ab	7.07 a	16.95	5.75 ab	3.27 b	−43.06
	**Total flavonol**	58.49 a	62.79 a	7.35	55.87 a	47.42 a	−15.14
2015	**Trisubstituted**						
	MyG	54.18 a	28.48 ** b	***−47.44***	45.24 a	28.54 ** b	***−36.92***
	**Disubstituted**						
	QcG	38.54 a	23.42 * b	***−39.08***	38.69 a	24.59 ** b	***−36.45***
	QcR	1.65 a	1.27 a	−40.50	1.71 a	1.34 a	−21.68
	QcGR	4.67 a	2.29 ** b	***−61.12***	3.95 a	2.17 ** b	***−44.96***
	QcGL	3.76 a	2.08 ** b	***−31.38***	3.47 a	2.13 * b	***−38.69***
	**∑Qc**	48.51a	29.70 * b	***−42.92***	47.80 a	30.22 ** b	***−36.37***
	IhG	4.27 a	2.45 ab	−78.59	2.45 ab	1.27 ** b	***−48.38***
	IhR	5.46 a	2.13 ** c	***−54.73***	5.21 ab	3.28 ** bc	***−37.04***
	**∑Ih**	9.73 a	4.59 ** b	***−65.27***	7.66 a	4.54 ** b	***−40.67***
	**Total disubstituted**	58.60 a	33.86 ** b	***−48.58***	55.71 a	35.12 ** b	***−36.96***
	***Monosubstituted***						
	KpG	4.60 a	2.39 ** b	***−48.12***	4.08 a	2.39 ** b	***−41.47***
	KpR	8.81 a	4.81 ** b	***−45.37***	8.93 a	5.11 ** b	***−42.84***
	***∑Kp***	13.41 a	7.20 **b	***−46.32***	13.01a	7.49 b	−42.41
	***Total flavonol***	126.31 a	69.98 *** b	***−51.31***	114.10a	71.95 *** b	***−36.94***
2016	***Trisubstituted***						
	MyG	49.73 a	21.47 *** c	***−56.83***	41.17 b	18.37 *** c	***−55.39***
	***Disubstituted***						
	QcG	27.63 a	16.84 ** b	***−39.08***	25.88 a	12.09 *** b	***−53.30***
	QcR	2.87 a	1.71 ** bc	***−40.50***	2.40 ab	1.26 ** c	***−47.52***
	QcGR	9.93 a	3.86 *** b	***−61.12***	8.42 a	3.06 *** b	***−63.65***
	QcGL	5.40 a	3.71 ** b	***−31.38***	4.98 a	2.45 *** c	***−50.85***
	**∑Qc**	45.83 a	26.11 ** b	***−42.92***	41.69 a	18.85 *** b	***−54.54***
	IhG	6.91 a	1.48 ** b	***−78.59***	5.35 a	3.04 *** b	***−43.23***
	IhR	8.73 a	3.95 ** c	***−54.73***	6.73 b	1.96 *** d	***−70.85***
	**∑Ih**	15.64 a	5.43 *** c	***−65.27***	12.07 b	5.00 *** c	***−58.61***
	**Total disubstituted**	61.83 a	31.79 *** b	***−48.58***	54.05 a	24.08 *** b	***−55.45***
	**Monosubstituted**						
	KpG	6.37 a	3.42 ** b	***−46.32***	5.25 a	3.84 ** b	***−26.89***
	KpR	4.44 a	2.57 ** b	***−42.02***	4.04 a	2.14 *** b	***−46.97***
	**∑Kp**	10.81 a	5.99 *** b	***−44.56***	9.29 a	5.98 ** b	***−35.62***
	**Total flavonol**	123.55 a	60.19 *** b	***−51.31***	105.44 a	49.23 *** b	***−53.23***

*, ** and *** indicate, for the same crop load (*H*/*L*), the significance of rainfed in respect to irrigation at *p* < 0.05, *p* < 0.01 and *p* < 0.001, respectively. For each parameter, means followed by different letters were significantly different between treatments, *p* < 0.05, Tukey’s HSD test.

**Table 6:** Influence of irrigation on concentration of flavanols compounds (mg. kg^−1^ berry fresh weight) under two crop load levels. *R*: rainfed; *I*: non-limiting irrigation; *H*: high crop load; *L*: low crop load. EGC: (−)-epigallocatechin; CA: (+)-catechin; CG: (−)-catechin gallate; EC: (−)-epicatechin; Pro B1, B2, B3 and A2: procyanidin B1, B2, B3 and A2.

Treatment							
Year	Compound	*RH*	*IH*	% Variation	*RL*	*IL*	% Variation
2014	**Catechins (CAT)**						
	ECG	41.18 b	36.70 b	−10.88	44.07 b	20.46 ** a	***−53.57***
	CA	6.70 a	10.70 a	59.70	7.64 a	11.46 * a	***50.00***
	CG	4.70 a	7.75 a	64.89	6.19 a	4.39 a	−29.08
	EC	5.52 a	7.40 a	34.06	5.62 a	8.10 ** a	44.13
	**Total catechins**	58.09 ab	62.54 ab	7.66	63.53 b	***44.41 a***	***−30.10***
	**Procyanidins (PRO)**						
	Pro B1	25.16 a	31.08 a	23.53	30.10 a	29.08 a	−3.39
	Pro B2	7.75 a	9.22 a	18.97	8.40 a	4.96 a	−40.95
	Pro B3	0.54 a	0.73 a	35.19	0.68 a	0.75 a	10.29
	Pro A2	0.65 a	0.81 a	24.62	0.86 a	2.02 a	134.88
	**Total procyanidins**	34.10 a	41.83 a	22.67	40.04 a	36.83 a	−8.02
	**Total flavanols**	92.19 a	104.37 a	13.21	103.57 a	81.24 a	−21.56
2015	**Catechins (CAT)**						
	ECG	37.36 a	40.03 a	7.15	34.75 a	34.36 a	−1.12
	CA	24.47 a	20.43 a	−16.51	22.10 a	17.90 a	−19.00
	CG	6.40 b	5.46 b	−14.69	1.18 a	5.91 ** b	400.85
	EC	9.72 a	8.14 a	−16.26	7.63 a	5.92 a	−22.41
	**Total catechins**	77.95 a	74.06 a	−4.99	65.65 a	64.09 a	−2.38
	**Procyanidins (PRO)**						
	Pro B1	37.43 a	35.95 a	−3.95	33.71 a	35.00 a	3.83
	Pro B2	13.32 a	11.99 a	−9.98	10.91 a	9.65 a	−11.55
	Pro B3	1.85 a	1.69 a	−8.65	1.75 a	1.63 a	−6.86
	Pro A2	0.75 a	0.53 a	−29.33	0.85 a	0.98 a	15.29
	Total procyanidins	53.36 a	50.16 a	−6.00	47.22 a	47.25 a	0.06
	**Total flavanols**	131.30 a	124.22 a	−5.39	112.87 a	111.34 a	−1.36
2016	**Catechins (CAT)**						
	ECG	57.49 c	41.56 * ab	***−27.71***	34.21 a	51.14 ** bc	***49.49***
	CA	27.59 a	31.97 a	15.88	33.91 a	21.62 * a	***−36.24***
	CG	10.39 ab	16.54 b	59.19	9.40 a	9.57 a	1.81
	EC	11.44 a	19.95 ** ab	74.39	20.86 b	11.59 * ab	−44.44
	**Total catechins**	106.92 a	110.01 a	2.89	98.39 a	93.91 a	−4.55
	**Procyanidins (PRO)**						
	Pro B1	151.02 b	79.70 *** a	***−47.23***	79.00 a	134.24 *** b	***69.92***
	Pro B2	18.17 a	18.37 a	1.10	19.08 a	13.26 a	−30.50
	Pro B3	2.99 a	2.66 a	−11.04	2.70 a	2.45 a	−9.26
	Pro A2	4.72 a	3.83 a	−18.86	3.79 a	4.17 a	10.03
	**Total procyanidin**	176.91 b	104.57 ** a	***−40.89***	104.57 a	154.12 *** b	47.38
	**Total flavanols**	283.83 b	214.58 * a	***−24.40***	202.96 a	248.03 * ab	*22.21*

*, ** and *** indicate, for the same crop load (*H*/*L*), the significance of rainfed in respect to irrigation at *p* < 0.05, *p* < 0.01 and *p* < 0.001, respectively. For each parameter, means followed by different letters were significantly different between treatments, *p* < 0.05, Tukey’s HSD test.

**Table 7:** Influence of irrigation on concentration of non-flavonoids compounds (mg. kg^−1^ berry fresh weight) under two crop load levels. *R*: rainfed; *I*: non-limiting irrigation; *H*: high crop load; *L*: low crop load. GA: gallic acid; CIN: cinnamic acid; FE: ferulic acid; CF: caffeic acid; CHL: chlorogenic acid; COU: *p*-coumaric acid; t-R: trans-resveratrol.

		Treatment					
Year	Compound	*RH*	*IH*	% Variation	*RL*	*IL*	% Variation
2014	**Hydroxybenzoic acids**						
	GA	3.06 a	3.19 a	4.25	2.56 a	2.60 a	1.56
	**Hydroxycinnamic acids**						
	CIN	11.41 ab	13.26 a	16.21	11.92 a	5.16 * b	***−56.71***
	FE	1.11 ab	1.30 ab	17.12	1.76 a	0.64 * b	−63.64
	CF	3.20 ab	3.40 ab	6.25	4.40 a	1.71 * b	***−61.14***
	CHL	3.07 a	3.58 a	16.61	4.57 a	2.94 a	−35.67
	COU	0.61 a	0.49 a	−19.67	1.12 a	0.56 * a	***−50.00***
	**Total hydroxycinnamic acids**	19.40 ab	22.03 ab	13.56	23.78 a	11.01 * b	***−53.70***
	**Stilbenes**						
	*t*-R	1.64 a	1.67 a	1.83	1.82 a	1.15 a	−36.81
2015	**Hydroxybenzoic acid**						
	GA	4.18 a	6.48 ** a	***55.02***	4.84 a	5.79 a	19.63
	**Hydroxycinnamic acids**						
	CIN	16.15 a	8.79 *** b	***−45.57***	15.03 a	7.99 ** b	***−46.84***
	FE	0.61 b	0.99 ** a	***62.30***	0.47 b	0.97 ** a	***106.38***
	CF	9.33 b	11.46 ** b	***22.83***	11.80 b	15.32 a	29.83
	CHL	0.99 b	0.44 ** b	***−55.56***	1.83 a	0.63 ** b	***−65.57***
	COU	0.89 ab	0.47 b	−47.19	1.16 a	0.33 ** b	***−71.55***
	**Total hydroxycinnamic acids**	27.97 ab	22.16 ** b	***−20.77***	30.30 a	25.24 ab	−16.70
	**Stilbenes**						
	*t*-R	1.09 a	0.65 ** b	***−40.37***	1.27 a	0.73 ** b	−42.52
2016	**Hydroxybenzoic acid**						
	GA	14.97 a	6.63 *** b	***−55.71***	14.28 a	6.95 *** b	−51.33
	**Hydroxycinnamic acids**						
	CIN	60.97 a	42.00 *** b	***−31.11***	48.42 b	31.12 ** c	***−35.73***
	FE	1.05 a	1.66 a	58.10	1.05 a	1.02 a	−2.86
	CF	34.62 a	29.46 * ab	***−14.90***	33.64 ab	28.52 * b	***−15.22***
	CHL	6.56 a	4.93 ab	−24.85	6.79 a	3.73 ** b	***−45.07***
	COU	11.44 a	5.57 *** b	***−51.31***	11.49 a	4.64 *** b	***−59.62***
	**Total hydroxycinnamic acids**	114.64 a	83.61 ** b	***−27.07***	101.39 a	69.02 ** b	***−31.93***
	**Stilbenes**						
	*t*-R	5.17 a	3.99 ** b	*−22.82*	4.12 b	3.42 * b	***−16.99***

*, **, *** indicate, for the same crop load (*H*/*L*), the significance of rainfed in respect to irrigation at *p* < 0.05, *p* <0.01, *p* < 0.001, respectively. For each parameter, means followed by different letters were significantly different between treatments, *p* < 0.05, Tukey’s HSD test.

## Data Availability

Not applicable.
